# Resident Training in Bariatric Surgery—A National Survey in the Netherlands

**DOI:** 10.1007/s11695-017-2729-z

**Published:** 2017-05-30

**Authors:** Gabrielle H. van Ramshorst, Mirjam A. Kaijser, Jean-Pierre E. N. Pierie, Bart A. van Wagensveld

**Affiliations:** 1 Present Address: VU University Medical Center, Amsterdam, the Netherlands; 2 The Netherlands Cancer Institute, P.O. Box 90203, 1006 BE Amsterdam, the Netherlands; 3 Department of Surgery, MCL Leeuwarden, Leeuwarden, the Netherlands; 4 PGSOM, UMCG/RU Groningen, Groningen, the Netherlands; 5 Department of Surgery, OLVG West, Amsterdam, the Netherlands

**Keywords:** Learning, Teaching, Bariatric surgery, Gastric bypass, Gastric sleeve, Coaching

## Abstract

**Purpose:**

Surgical procedures for morbid obesity, including laparoscopic Roux-en-Y gastric bypass (LRYGB), are considered standardized laparoscopic procedures. Our goal was to determine how bariatric surgery is trained in the Netherlands.

**Materials and Methods:**

Questionnaires were sent to lead surgeons from all 19 bariatric centers in the Netherlands. At least two residents or fellows were surveyed for each center. Dutch residents are required to collect at least 20 electronic Objective Standard Assessment of Technical Skills (OSATS) observations per year, which include the level of supervision needed for specific procedures. Centers without resident accreditation were excluded.

**Results:**

All 19 surgeons responded (100%). Answers from respondents who worked at teaching hospitals with residency accreditation (12/19, 63%) were analyzed. The average number of trained residents or fellows was 14 (range 3–33). Preferred procedures were LRYGB (*n* = 10), laparoscopic gastric sleeve (LGS) resection (*n* = 1), or no preference (*n* = 1). Three groups could be discerned for the order in which procedural steps were trained: unstructured, in order of increasing difficulty, or in order of chronology. Questionnaire response was 79% (19/24) for residents and 73% (8/11) for fellows. On average, residents started training in bariatric surgery in postgraduate year (PGY) 4 (range 0–5). The median number of bariatric procedures performed was 40 for residents (range 0–148) and 220 during fellowships (range 5–306).

**Conclusions:**

Training in bariatric surgery differs considerably among centers. A structured program incorporating background knowledge, step-wise technical skills training, and life-long learning should enhance efficient training in bariatric teaching centers without affecting quality or patient safety.

## Introduction/Purpose

Bariatric surgery has become a substantial part of the workload of gastrointestinal surgeons [1]. In the USA, residents who have completed surgical training can apply for fellowships accredited by the American Society for Metabolic and Bariatric Surgery (ASMBS) [2, 3] In Europe, resident programs and postgraduate training differ per country. In the Netherlands, bariatric surgery is performed in 19 high-volume centers, most of which are non-academic teaching hospitals. In some of these centers, unaccredited bariatric fellowships are offered, usually on an irregular basis*.* According to the Dutch guidelines for bariatric surgery, centers can only be certified if at least 200 bariatric procedures are performed by a minimum of two surgeons, with each surgeon performing at least 30 procedures [4]. The general surgery residency is a 6-year program, which included 1 to 2 years of training at academic teaching hospitals. Track specialization as proposed by Martin et al. has been incorporated in the Dutch curriculum [5]. Residents are obliged to choose one subspecialty for the final 2 years of training: gastrointestinal surgery (including bariatric surgery as an option), oncological surgery, vascular surgery, traumatology (including orthopedic trauma surgery), lung surgery, or pediatric surgery.

Surgical procedures for morbid obesity, including laparoscopic Roux-en-Y gastric bypass (LRYGB) and laparoscopic gastric sleeve resection (LGS), can be considered standardized laparoscopic procedures [4]. Therefore, these procedures can be considered ideal for structured teaching of laparoscopic surgery skills to residents [1]. However, large differences exist in the skill levels that residents reach in advanced laparoscopic and bariatric surgery, with a varying level of required supervision after completion of residency training [6]. This study aims to determine how residents and fellows are trained in bariatric surgery in the Netherlands.

## Materials and Methods

Questionnaires were sent to lead surgeons from all 19 bariatric centers in the Netherlands by email and/or by regular mail in November 2014. Surgeon data included age, gender, number of years of experience with performing bariatric surgery, hospital setting (private practice, non-academic teaching hospital, non-academic non-teaching hospital, or academic hospital), and yearly number of bariatric procedures. Also, surgeons were asked which was the preferred training procedure for residents (LRYGB/LGS), which pre-clinical courses or other type of instruction prepared residents before performing bariatric surgery, and the level of supervision reached by the residents at the end of formal training (A = assisting, B = strict supervision, C = limited supervision, D = without supervision, E = supervising). Surgeons were also interviewed on relevant technical details, such as the number of trocars used in LRYGB (excluding the liver retractor). Finally, the postgraduate year in which residents were allowed to perform specific steps of LRYGB and LGS was recorded.

At least two residents or fellows were surveyed for each center. Questionnaires were sent by email and regular mail. Residents and fellows were asked the following data: age, gender, postgraduate year (PGY), track specialization, and hospital setting. The survey also consisted of questions on how many bariatric operations they had performed, the level of supervision, and in which order and pace different steps were instructed. Dutch residents are required to collect at least 20 electronic Objective Standard Assessment of Technical Skills (OSATS) observations per year, which include the level of supervision needed for specific procedures. The full list of translated questions is stated in the Appendix. Reminders were sent after 2 and 6 weeks. In February 2015, the survey was closed.

For analyses, data from residency-accredited hospitals were included. Data from centers with unaccredited fellowships without residency accreditation were excluded.

## Results

### Surgeons’ Responses

Surgeons from all 19 centers participated in the study (100%). Baseline data of the 19 lead surgeons and residency accreditation are summarized in Table 1. The answers from 12 respondents working in resident teaching centers (12/19 = 63%) were analyzed in more detail. In these 12 hospitals, the average number of trained residents or fellows was 14 (range 3–33). LRYGB was the preferred training procedure in 10 centers, laparoscopic sleeve resection in 1 center, and in 1 center both procedures were taught equally. A mean number of four trocars were used (range three–five). Lead surgeons, fellows, and residents reported the PGY in which residents started performing various steps of the procedures (Table 2).

**Table 1:** Baseline characteristics of surgeons and hospitals

	Non-teaching hospital	Teaching hospital
Gender (male/female)	6:1	11:1
Age (years)	48 (38–62)	50 (38–62)
Experience (years)	9 (5–12)	12 (4–25)
No. of bariatric cases/year	273 (120–400)	235 (100–350)
No. of trained residents	–	10 (2–22)
No. of trained fellows	0.4 (0–3)	6 (0–33)
Total no.	0.4 (0–3)	15 (3–33)

Data displayed as mean (range)

**Table 2:** Reported average start of postgraduate training year for each step of laparoscopic gastric bypass and gastric sleeve procedure as reported by lead bariatric surgeons

	Gastric bypass		Gastric sleeve	
	Reported PGY		Reported PGY	
Pre-operative checklist				
Instrument check	1.5	(1–4)	1.5	(1–4)
Team position	1.5	(1–4)	2	(1–4)
Monitor position	2	(1–4)	2	(1–4)
Patient position	2	(1–4)	2	(1–4)
Antibiotic prophylaxis check	1	(1–4)	2	(1–4)
Disinfection/sterile field	1	(1–4)	2	(1–4)
Insertion of gastric bougie	1.5	(1–4)	2	(1–4)
Initializing pneumoperitoneum				
Camera-assisted trocar introduction	2	(1–4)	2	(1–4)
Introduction of Veress needle	2	(1–4)	2	(1–4)
Introduction of trocars	2	(1–4)	2	(1–4)
Diagnostic laparoscopy	2	(1–4)	2	(1–4)
Exposure operating field				
Introduction of liver retractor	2.5	(1–5)	3	(1–5.5)
Opening of pars flaccid	3	(1–4)	–	–
Exposition of greater curvature	–	–	3	(2–6.5)
Exposition of crus	–	–	3.5	(2–6.5)
Stapling of stomach/sleeve	4	(2–5.5)	3.5	(2–6.5)
Separation of greater omentum	3.5	(2–5.5)	–	–
Measuring jejunum from Treitz	4	(2–6)	–	–
Stapled gastro-enterostomy	4	(2–6)	–	–
Placement of stay sutures	3.5	(3–5.5)	–	–
Suturing pouch defect	4	(2–6)	–	–
Measuring jejunum pouch-distal	3.5	(2–6)	–	–
Creation of stapled jejuno-jejunostomy	4	(2–6)	–	–
Placement of stay sutures on jejunum	2	(2–5.5)	–	–
Suture of jejunal anastomotic defect	4	(2–6)	–	–
Transection of the bowel limb	4	(1–6)	–	–
Closure of mesenteric defect	4	(1–6)	–	–
Extraction of gastric remnant	–	–	3.5	(2–6.5)
Finishing procedure				
Removal of gastric bougie	2	(1–4)	2	(0–4)
Removal of liver retractor	2.5	(1–4)	2	(1–4)
Removal of trocars	2	(1–4)	2	(1–4)
Closing fascia defects >10 mm	2	(1–4)	2.5	(1–4)
Skin suturing	1	(1–2)	1	(1–3)

Data displayed as median (range). Fellowship training is displayed as PGY 7 to 8

The majority of lead surgeons stated that residents should be experienced with basic laparoscopic procedures (appendectomy, cholecystectomy, inguinal hernia repair) before embarking on bariatric procedures. All surgeons mandated completion of a basic laparoscopic training course (100%). Also, some surgeons actively reported that residents should show clear interest in the procedures and the background of metabolic surgery. One of the lead bariatric surgeons reported giving supervised laparoscopic box training before starting in vivo training. At the end of residency or fellowship training, 16% of residents and 66% of fellows were able to perform bariatric surgery independently and/or to supervise other fellows or residents (Table 3).

**Table 3:** Postgraduate year (PGY) training levels and numbers of procedures performed as reported by surveyed residents and fellows

Type of procedure	Residents				Fellows							
					Residency				Fellowship			
	Procedures (*n*)		Skill level		Procedures (*n*)		Skill level		Procedures (*n*)		Skill level	
LRYGB	20	(0–100)	C	(A–E)	38	(0–200)	D	(B–D)	150	(5–375)	D	(B/C–D/E)
Single anastomosis gastric bypass	0	(0–30)	C	(A–D)	–		–	–	0	(0–18)	–	–
Lap gastric sleeve	8	(0–40)	C	(B–E)	14	(0–60)	D	(D)	14	(5–51)	D	(D–E)
Open RYGB	0	(0–5)	B	(A–D)	–		–	–	0	(0–1)	D	(D)
Lap gastric banding	0	(0–10)	A	(A)	18	(0–50)	–	–	5	(0–50)	–	–
Lap band removal	4	(0–20)	C	(A–D)	0	(0–100)	D	(C–D)	16	(3–70)	D	(D)
Redo procedures	0	(0–30)	D	(B–E)	0	(0–15)	E	(D–E)	11	(0–75)	D	(D)
Duodenal switch	0	(0–1)	B	(A–D)	0	(0–21)	D	(C–D)	0	(0–5)	D	(D–D.E)
Total	40	(0–148)			93	(25–275)	–	–	220	(5–306)	–	–

Data are displayed as median (range)

*(L)RYGB* (laparoscopic) Roux-en-Y gastric bypass, skill level: *A* assisting, *B* under strict supervision, *C* under limited supervision, *D* without supervision, *E* supervising

### Teaching Methods

Three main groups could be discerned for the order in which procedural steps were taught:

1. Unstructured (*n* = 3)
2. Increasing difficulty—entero-enterostomy, pouch, gastro-enterostomy (*n* = 6)
3. Chronology—pouch, gastro-enterostomy, entero-enterostomy (*n* = 3)

In the unstructured training group, residents started performing the procedure or parts of the procedure without a clear training plan or structure. In the second group, handling of the stapler and intracorporal suturing were practiced first during creation of the entero-enterostomy and later in creation of the pouch and gastro-enterostomy. Centers who had trained high numbers of residents/fellows were more likely to use this training order. In the third group, steps were taken in chronological order with creation of the pouch, gastro-enterostomy, and finally, the entero-enterostomy.

One of the surveyed hospitals reported on their previously published training model in detail [6]. Stringent pre-surgery conditions applied, including an advanced laparoscopic suturing course and performance of 100 basic laparoscopic procedures such as cholecystectomy and appendectomy. Residents commenced with assisting 10 LRYGB procedures. Next, the residents performed the first teaching step of the procedure, the distal anastomosis, in subsequent operations until this step was mastered, meeting the standards of both bariatric surgeons in this bariatric center. Next, the second teaching step, creating the pouch, was practiced to complete. The third and final teaching step was creation of the gastro-enterostomy. Supervised laparoscopic box training was offered on a regular basis. To avoid prolongation of operating times, only after sufficient skills on all three steps, residents were allowed to integrate the steps into the full procedure. Surgeons reported that the results of this training technique results showed that residents could be taught the full procedure without significant increase in duration of surgery or complications of the LRYGB [6].

### Resident and Fellow Responses

Overall questionnaire response was 79% (19/24) for residents, with a mean age of 34 years (range 30–39 years) and 73% (8/11) fellows, with a mean age of 36 years (range 34–39 years). Most residents started bariatric surgery in PGY 4 (range 0–6 years) and were female (11/19). One resident had started assisting and performing laparoscopic bariatric surgery during his internship (defined as PGY 0). Fellows had completed general surgical training 14 months before the survey (range 3–28 months) and were predominantly male (7/8). Fellows had started performing bariatric surgery in PGY 5 (range 2–6).

The median number of bariatric procedures performed was 40 for residents (range 0–148) and 220 during fellowships (range 5–306, (Table 3). The median number of assisted procedures was 52 (range 8–1100). Residents performed a median of 20 LRYGB procedures during their residency. Fellows had performed a median of 38 LRYGB during their residency and 150 LRYGB during their fellowships. The single anastomosis gastric bypass was only performed by a small minority of residents and fellows. LGS was performed 8 times during residency, and 14 times during the residencies of those working as fellows (median). During fellowships, a median number of 14 (range 5–51) LGS procedures were performed. Open procedures of gastric bypass and duodenal switch were rare (*n* = 5 and *n* = 1, respectively).

At the time of interview, most residents had reached level of supervision C (performing surgery under limited supervision). One resident had reached level A (assisting; 5%), three level B (strict supervision, 16%), 12 level C (limited supervision, 63%), two level D (without supervision, 11%), and one was supervising LRYGB (5%). Six out of eight fellows were able to perform the operations without supervision (75%), one as supervisor (12.5%), and one performed LRYGB under limited supervision (12.5%). Eight residents were able to perform LGS resections without supervision.

### Scientific Meetings and Courses

Six respondents reported having visited meetings of the Dutch Society for Metabolic and Bariatric Surgery (DSMBS), and nine respondents had joined congresses of the International Federation for the Surgery of Obesity and metabolic disorders (IFSO). Other reported meetings were advanced laparoscopic suturing courses and medical industry-driven symposia. Seven respondents had not participated in any scientific meeting dedicated to bariatric surgery (26%).

### Out Patient Clinic and National Training

A majority of residents and fellows (78%, 21/27) reported regular involvement with bariatric patients in the outpatient clinic, with six respondents actively reported seeing new patients (22%), and five respondents had performed follow-up of operated patients (19%).

Half of respondents (13/27) believed that bariatric surgery should be a voluntary topic in the national resident training program’s lectures, 12 trainees believed that this should be a mandatory subject, and the remaining two respondents had no opinion.

## Discussion

Our survey is the first national survey on resident training in bariatric surgery. Teaching experience varied highly between centers. In centers with more teaching experience, training programs were developed and were more likely to teach residents/fellows procedural steps in order of increasing difficulty. All residents who started performing bariatric procedures were experienced with basic laparoscopic procedures. The number of assisted procedures showed high variability among residents. The use of more than three trocars could be beneficial for residents/fellows, as this allows for giving and receiving of assistance in laparoscopy (“a helping hand”) as part of the learning experience. One resident had assisted in 1100 laparoscopic bariatric procedures (mainly gastric band placement) as part of preparation for his PhD thesis on bariatric surgery. The fellows had performed more procedures during their residency than the current residents, which may be reflective of selection bias. One resident and three fellows reported a case load of more than 100 procedures during residency, and four residents more than 50 procedures. This suggests that it is possible to overcome the supposed learning curve of the LRYGB during residency. For those with less experience, either a fellowship or supervised proctoring will still be needed to pursue a career in bariatric surgery. In our survey among (former) fellows, five out of eight fellows had performed over 200 procedures, with a significant number of these procedures performed without a supervising attendant. The presence of a supervising attendant was not recorded for residents, which hindered the exact interpretation of training level D “without supervision.”

The effects of resident and fellow involvement in bariatric surgery on operation times and, most importantly, patient safety outcomes, have been discussed in several publications [7–11]. In a retrospective study on 17,057 LRYGB patients from Michigan, resident involvement was an independent risk factor for wound infection, but not for venous thromboembolism [7]. Several analyses of patients included in the American College of Surgeons NSQIP database have been published. Davis et al. reported a series of 12,390 LRYGB patients, showing that resident involvement was associated with increased morbidity rates (4.0 vs. 5.2%, *p* < 0.01) [8]. In another cohort of 10,838 LRYGB and gastric sleeve resection patients, fellow involvement was found to be an independent risk factor for complications (overall, serious, and surgical) and reoperation rates in the LRYGB group, but not for gastric sleeve resection [9]. During the first 6 months of fellowship, Bhayani et al. specifically noted increased rates of surgical site infection, urinary tract infection, deep veinous thrombosis, and sepsis [10]. After 6 months, outcomes were similar to patients operated by attending. On the other hand, a multivariate analysis of a database of 47,342 patients from New York State showed that bariatric fellowship accreditation was significantly associated with improved perioperative patient outcomes compared to non-fellowship-accredited hospitals [11]. Mortality was not associated with fellow participation in any of the aforementioned studies [7–11]. These data support the continued need for training programs for both residents and fellows to improve technical skills.

The previously described teaching model incorporated partially supervised laparoscopic box training with an efficient program to teach residents using an in vivo step-by-step method [6]. The surgeons from this center chose to teach the steps in order of increasing difficulty, which is consistent with contemporary teaching principles [12]. The division of the procedures into several steps is thought to be a result of time pressure of the operating schedule. Several studies have supported breaking up procedures into sub-steps to facilitate training in vivo or in a training laboratory [13–15]. Description of procedural key steps, which has been performed for laparoscopic colorectal surgery, cholecystectomy, and appendectomy, can help to establish structured teaching programs [16, 17]. We intend to describe these key steps for LRYGB and LGS in the future.

It is noteworthy that the involvement of residents in the outpatient clinic for follow-up of patients and complications proved limited. As future surgeons, residents need to be able to actively inform patients on the risks and benefits of bariatric surgery, but should also be instructed to perform adequate follow-up, detect, and treat complications of bariatric surgery. As proposed by Schirmer et al., dedicated fellowship programs should include journal clubs and teaching rounds and provide the opportunity to contribute to research [18].

Currently, the effectiveness of most different training methods for bariatric surgery has remained unclear. Based on the outcomes of our survey and the available data in literature [12, 19, 20], we recommend the following components for a training program in bariatric surgery:

1. Background of bariatric and metabolic surgery

A variety of methods (e-learning, courses, video instructions) can be used to instruct residents on patient selection, pathophysiology, operative procedures, and complication management. Multidisciplinary conferences and morbidity and mortality conferences should be considered part of the training.

2. Technical skill training

- Ex vivo laparoscopic dexterity training (unsupervised box training and supervised box training with formative feedback)
- Cadaver training for specific procedures
- Step-wise supervised in vivo training in selected patients

Surgical coaching and regular feedback using bariatric OSATS, Global Operative Assessment of Laparoscopic Skills (GOALS), and an independence-scaled procedural assessment should be provided [21]. Key steps should preferably be determined using Delphi consensus and mastered in level of increasing difficulty before continuation to the next step. Individual steps should be combined to result into performance of full procedures, and further development of skills according to the Dreyfus and Dreyfus model [19].

3. Life-long learning

Continuous medical learning can be achieved through participation in journal clubs, teaching rounds, clinical research, and (inter-)national scientific meetings. Also, reporting surgical outcome data in national databases is important for establishing benchmarks and quality improvement.

General recommendations for professional and personal growth such as team training, self-assessment, and peer assessment were not included in this proposal, as these should preferably be integrated in a personal professional development plan.

Limitations of our study include the low number of fellows who participated in our survey, which is reflective of the lack of accreditation for bariatric fellowships in the Netherlands. Also, based on the overall low number of LGS procedures nationwide, no data on learning steps of this procedure were available. Our study showed that bariatric procedures can be trained during residency in our country, and supports the statement of Eltorai that bariatric surgery could be incorporated in the resident training curriculum [22]. It may, however, be difficult to extrapolate our study results and recommendations to other countries where different training models are used. European working hour regulations have become strict, with 48-h working weeks probably resulting in higher absence of residents in operating theaters and in outpatient clinics. Moreover, shortening of medical doctor specialist training has been advocated, as for example in the UK [23]. Restriction of resident working hours in the USA to 80 h per week has impacted resident exposure to complex cases and overall presence, as summarized by Varas [24]. Therefore, it is essential that residents are trained efficiently, while ensuring patient safety. We believe that our recommendations may help to use the period of structured training in patients more effectively.

## Conclusion

Training of residents and fellows in bariatric surgery differs considerably among centers. A structured program incorporating background knowledge, step-wise supervised technical skill training, and life-long learning should enhance efficient training in bariatric teaching centers without affecting quality or safety.

## Appendix– Resident Questionnaire

- In which year of postgraduate training did you start with bariatric surgery and how was this training built up in terms of procedures?
- Report the number and type of bariatric procedures performed and the level of supervision needed for the following procedures: laparoscopic gastric bypass, laparoscopic single anastomosis gastric bypass, laparoscopic gastric sleeve resection, open gastric bypass (including redo after failed banding), open gastric sleeve resection, laparoscopic gastric banding, laparoscopic removal of gastric banding, laparoscopic removal of gastric banding and simultaneous gastric bypass, duodenal switch or other procedure (Table provided)
- Describe the structure of your learning process of the laparoscopic gastric bypass, using the provided table with procedural steps if necessary.
- To which extent was the outpatient clinic for bariatric surgery part of your training? (How often did you attend? Was supervision available / accessible)?
- Which courses and / or training did you complete in this field before you started performing bariatric surgery? (E.g., basic laparoscopy course, visit bariatric congress / pre-course program, research, presentation, assisted procedures)
- Which of the aforementioned courses / programs were mandated by the program director and / or reimbursed financially?
- Which courses / training you have completed since you started with bariatric surgery? (E.g.: visit bariatric congress / pre-course program, research, presentation)
- Which of the aforementioned courses / programs were mandated by the program director and / or reimbursed financially?
- Did you miss any courses / programs? If so, what have you missed and at what point would this have fitted into your training?
- Do you think bariatric surgery should be taught as part of the surgical training program lectures? Should it be mandatory or optional? What is the ideal length of the course? Is practical training needed?
